# Comparing the Efficacy of Topical 4% Benzoyl Peroxide Versus Topical 0.1% Adapalene for Treatment of Acne Vulgaris in Skin of Color Population: A South Asian Perspective

**DOI:** 10.7759/cureus.55555

**Published:** 2024-03-05

**Authors:** Asmat Ullah, Anjum Muhammad, Farman Mehmood, Hina Farooq, Bilal Ahmad, Afnan Bin Haq, Naseem Khan, Syeda Naz, Asghar Khan, Afshan Saeed

**Affiliations:** 1 Dermatology, Pak-Emirates Military Hospital, Rawalpindi, PAK; 2 Plastic Surgery, Combined Military Hospital, Rawalpindi, PAK; 3 Endocrinology and Diabetes, Pak-Emirates Military Hospital, Rawalpindi, PAK; 4 Dermatology, Combined Military Hospital, Gujranwala, PAK; 5 Medicine, Khyber Medical University, Peshawar, PAK

**Keywords:** acne treatment india and pakistan, skin of color, skin of color dermatology, adapalene efficacy, benzoyl peroxide, adapalene, acne

## Abstract

Introduction

Acne vulgaris is one of the most common skin problems encountered in the dermatology department. It is a chronic, inflammatory disease of the pilosebaceous unit, clinically presenting with comedones, papules, pustules, nodules, and cysts. With its particularly high prevalence in the younger population, it has significant adverse sequelae on patient’s quality of life. At present, due to an enhanced understanding of the pathogenesis of acne, various therapeutic modalities are available. The current management strategies generally follow a systematic treatment escalation based on disease severity and treatment response. However meticulous choice of appropriate anti-acne medicine for the acne type is the key to the management plan. Starting with mild to moderate types of acne as per the Leeds photometric grading scale, the most useful topical agents include topical retinoids, benzoyl peroxide, and topical antibiotics while systemic therapies such as oral antibiotics or isotretinoin are generally reserved for moderate to severe acne treatment. The skin of color (SOC) population is a relatively neglected group concerning the optimum and safe management strategies in different dermatological conditions and acne is no different, where there remains a need for comparing the available topical modalities for appropriate drug selection in the treatment of mild to moderate acne in SOC population.

Objective

The objective of this study was to compare the efficacy of topical 4% benzoyl peroxide versus topical 0.1% adapalene in the treatment of acne vulgaris in the SOC population.

Methods

The participants were divided into two groups, groups A and B. A total of 64 patients of both genders, with acne vulgaris (duration > three months) were included in the study. In group A, 32 patients were administered topical 0.1% adapalene whereas, in group B, 32 patients were given topical 4% benzoyl peroxide. Both medicines were applied at night daily. Patients were called for follow-up after 12 weeks. In both groups, the final efficacy evaluation was done using the Global Acne Grading System (GAGS) score after 12 weeks of treatment period.

Results

In group A, the age ranged from 15 to 40 years with a mean age of 25.781±3.93 years while the duration of complaint was 5.843±1.27 months. GAGS score was 25.281±2.65 and mean BMI was 23.092±3.51 kg/m2. In group B, the mean age was 25.187± 4.06 years, the duration of complaint was 7.375±2.25 months, the GAGS score was 23.906± 2.60 while the mean BMI was 21.485±3.88 kg/m2. Efficacy in group A was noted in 25 (78.1%) patients as compared to 24 (75%) patients in group B (p =0.768).

Conclusion

The present study showed that the safety and efficacy of 0.1% adapalene the traditional drug 4% benzoyl peroxide in the SOC population was comparable.

## Introduction

Acne vulgaris is a chronic, inflammatory condition of the pilosebaceous glands that impacts 34.3-95% of juveniles and adolescents aged nine to 18 years old, making it the most common dermatological ailment of the above-mentioned age group [1,2]. Clinically, acne appears as a combination of non-inflammatory (open or closed comedones) and inflammatory lesions (papules, pustules, nodules, cysts) on areas of the skin with abundant sebaceous glands, such as the face, chest, and upper back. Albeit self-limiting, acne can take many years to settle imposing a considerable burden on those affected, as it has been connected to entities like anxiety, low self-esteem, depression, and diminished quality of life (QoL), irrespective of the span and severity of acne or age of onset [1].

Early treatment is encouraged as up to 43% of acne patients may develop permanent scars and hyperpigmentation due to prolonged uncontrolled acne lesions [2]. Acne treatment targets four mechanisms: sebaceous gland hyperplasia, abnormal follicular growth, Propionibacterium acnes colonization, and innate immunity activation. Effective treatment employs multiple medications to address at least two of these causes [1,3]. The current strategies of acne management involve treatment escalation based on disease severity as assessed by the Leeds photometric grading scale and the treatment response. Leeds photometric grading scale for acne stratifies acne from grades I-IV, with grade I being mild ( predominantly comedones, with <10 small papules and pustules), Grade-II as moderate (with 10-40 papules and pustules), grade III as moderate-severe (40-100 papules and pustules with few nodules) and grade-IV as severe acne (with nodulocystic and conglobate lesion with pain along with papules, pustules and comedones) [4]. Topical treatments like retinoids, benzoyl peroxide, and topical antibiotics are used first. If these are not effective, oral antibiotics or isotretinoin may be prescribed [5]. Yet, because of the adverse effects like irritation, systemic toxicity, bacterial resistance, and the chronicity of the disease, novel remedies that target the various pathogenic pathways of acne with minimal side effects are still alluring.

The skin of color (SOC) population is a relatively neglected group concerning the knowledge of the presentation of various diseases and optimum and safe management strategies in different dermatological conditions [6,7]. The situation in the case of acne is largely similar where the relative lack of adequate data regarding the comparative efficacy and side effect profile on the already established medications in the SOC population makes the situation more perplexing [8]. A limited number of studies have been conducted that compare the efficacy and safety of certain topical retinoids in the SOC population [9]. The amount of work done on the comparative efficacy of other drugs such as adapalene and benzoyl peroxide in the SOC population is even more limited. Of whatever previous data reported by local studies on the SOC population, the results have largely been contradictory [10]. Therefore, it seemed worthwhile to conduct a study on the SOC population to ascertain the comparative efficacy of these drugs which may help decide the appropriate drug selection for the effective management of acne in such patients.

## Materials and methods

This quasi-experimental study was carried out in the dermatology department of a tertiary healthcare facility in Rawalpindi Pakistan, from October 10, 2021, to April 10, 2022, after due approval from the ethical committee of the institution (A/28/PEMH/21/EC/03). Non-probability consecutive sampling technique was employed in the study and sample size was calculated using the WHO calculator, with a 95% confidence level with the power of the study being 90%. By using the expected proportion, (efficacy of topical 0.1% adapalene by 78% and efficacy of 4% benzoyl peroxide by 30%) [11] estimated sample size came out to be n=32 for 0.1% adapalene Group (Group A), while n=32 for 4% benzoyl peroxide group (Group B). Informed consent was acquired from all the participants of the study.

Patients aged 15-40 years of both genders, with Fitzpatrick type III and above skin grades, suffering from acne vulgaris, as per operational definition, of duration > three months and a Global Acne Grading System (GAGS) score < 30 (Mild to Moderate) were included in the study. (The GAGS score is composed of three categories: 1-18 is mild acne, 19-30 is moderate acne, 31-38 is severe acne, and above 39 is very severe acne. The GAGS score calculation is mentioned in the lower part of the special proforma as shown in the Appendices.) Those with a previous history of topical therapy in the last two months, a history of using estrogens/birth control pills, positive pregnancy on laboratory tests, and any chronic comorbidities that may affect the treatment outcomes were excluded from the study. Fitzpatrick type II and I grade and those unable to maintain a follow-up were excluded from the study.

Sixty-four patients fulfilling the inclusion criteria from the department of dermatology were included in the study. Randomization was conducted through sequentially numbered opaque envelopes generated from a random numbers table into two groups of 32 patients each. Each patient was assigned a number at enrolment which defined a study drug assignment (0.1% adapalene or 4% benzoyl peroxide). In group A, 32 patients were administered topical 0.1% adapalene, while in group B, 32 patients were prescribed topical 4% benzoyl peroxide, to be applied at night on a daily basis in both groups. Patients were called for follow-up after 12 weeks. The final assessment of efficacy (as per operational definition) was carried out at the end of 12 weeks of treatment duration in both group A and group B. Efficacy was noted by trained researchers on a specially designed proforma (see Appendices) that noted age, BMI, height, weight, GAGS score at the start and end of the study, and efficacy of both agents. The salient side effects of both drugs were also recorded on subsequent visits.

Data was analyzed with the statistical analysis software program Statistical Package for Social Sciences (SPSS), version 23.0 (IBM Corp., Armonk, NY). Mean and standard deviation (SD) were presented for quantitative variables like age, duration of complaint, GAGS score, and BMI. Frequency and percentages were computed for qualitative variables like age groups, gender, and efficacy. The chi-square test was applied to compare efficacy in both groups with a p-value of ≤0.05 taken as significant.

## Results

In our study, the age ranged from 15-40 years. In group A, the mean age was 25.781±3.93 years, the duration of complaint was 5.843±1.27 months, the GAGS score of 25.281±2.65 pre-therapy, the mean BMI was 23.092±3.51 kg/m2. While in group B, the mean age was 25.18±4.06 years, the duration of complaint was 7.375±2.25 months, the pre-treatment GAGS score was 23.906±2.60 and the mean BMI was 21.485 ± 3.88 kg/m2 as shown in Table 1.

**Table 1:** Age, duration of complaints, GAGS score, and BMI in both groups, A and B (n=64) GAGS: Global Acne Grading System

Demographics	Mean±SD Group A n=32	Mean±SD Group B n=32
Age(years)	25.781±3.93	25.187±4.06
Duration of complaint (months)	5.843±1.27	7.375±2.25
GAGS score	25.281±2.65	23.906±2.60
BMI (kg/m2)	23.092±3.51	21.485±3.88

Around two-thirds (65.6%, n=21) of the patients in both groups were female. Efficacy was seen in n=25 (78.1%) patients in group A as compared to n=24 (75%) patients in group B (p =0.768) as shown in Table 2. The results showed that there was no difference in both groups in terms of their efficacy in the SOC population.

**Table 2:** Comparison of efficacy in both groups, A and B (n=64); chi-square analysis is done

Group	Efficacy, n (%)		p-value
	Yes	No	0.768
A	25 (78.1%)	7 (21.9%)	
B	24 (75%)	8 (25%)	

Stratification of efficacy in both groups concerning age, gender, duration of complaint, GAGS score, and BMI as shown in Table 3 also suggested that adapalene and benzoyl peroxide showed similar efficacy when the efficacy of the drugs was observed with regard to age and gender of the patients as well as the duration and severity of the acne. Similarly, both drugs were well tolerated and neither of the two, led to discontinuation of the drug in any patients.

**Table 3:** Stratification of efficacy concerning age, gender, duration of complaints, GAGS score, and BMI (n=64); chi-square analysis is done GAGS: Global Acne Grading System

Age (years)	Groups	Efficacy, n (%)		p-value
		Yes	No	
15-30	A	22 (81.5%)	5 (18.5%)	0.380
	B	20 (71.4%)	8 (28.6%)	
31-40	A	3 (60%)	2 (40%)	0.138
	B	4 (100%)	0 (0%)	
Gender				
Male	A	10(90.9%)	1(9.1%)	1
	B	10(90.9%)	1(9.1%)	
Female	A	15 (71.4%)	6 (28.8%)	0.738
	B	14 (66.7%)	7 (33.3%)	
Duration of Complaints				
3-6 months	A	17 (77.3%)	5 (22.7%)	0.783
	B	11 (73.3%)	4 (26.7%)	
>6 months	A	8 (80%)	2 (20%)	0.831
	B	13 (76.5%)	4 (23.5%)	
GAGS Score				
19-25	A	16 (100%)	0 (0%)	0.225
	B	21 (91.3%)	2 (8.7%)	
>25	A	9 (56.2%)	7 (43.8%)	0.270
	B	3 (33.3%)	6 (66.67%)	
BMI				
≤ 25	A	21 (80.8%)	5 (19.2%)	0.466
	B	21 (72.4%)	8 (27.6%)	
>25	A	4 (66.67%)	2 (33.33%)	0.256
	B	3 (100%)	0 (0%)	

## Discussion

The study was devised to compare and assess the effectiveness of adapalene, a third-generation retinoid, and the traditional drug benzoyl peroxide SOC population. There is a relative scarcity of data regarding the optimal and safe management strategies of different dermatological illnesses in the SOC population, a fact evident by various clinical trials performed in the West, where the SOC population is largely underrepresented compared to their actual population proportion [8,12]. This, in part, can be explained by the relatively poor access to quality healthcare services and research facilities, of the SOC population, both in the West and their native countries [12].

Acne being one of the most common dermatological ailments, possesses a similar profile in the SOC population, where the comparative safety and efficacy of different in-vogue treatment modalities is relatively scarcely reported, hence leaving the demands of a huge population, largely unmet. Topical agents like benzoyl peroxide and retinoids are preferred for long-term use due to their safety compared to other treatment methods like oral antibiotics and retinoids. The latter are known to cause adverse reactions such as bacterial resistance and other side effects due to their absorption into the systemic circulation. The results of our study confirm the comparable efficacy of 0.1% adapalene and 4% benzoyl peroxide in acne vulgaris in the SOC population, thus filling the knowledge gap for better management of acne in such a population. Both medications fetched prompt clinical therapy and demonstrated identical in terms of effectiveness and tolerability.

Most of the patients were in the 15-30 years age group (Table 1), corroborating the findings reported by a large systemic analysis regarding the worldwide trends on multiple epidemiological variables of acne [13]. This is probably because androgenic hormones are at their peak in this age group, causing sebaceous gland stimulation due to hormonal surge during puberty. Overactive sebaceous glands in the skin and a build-up of oil, dead skin cells, and bacteria lead to inflammation in sebaceous ducts. A higher preponderance of women was recorded as compared to males in our analysis. This observation was also comparable to international trends [13] and can be attributed to the verity that women are more cognizant of their cosmetic disfigurement and care to show earlier at dermatology clinics than their male counterparts. Adapalene, in our study, demonstrated outstanding effectiveness in 78.1% of our subjects. Albeit effective in both inflammatory and non-inflammatory lesions, the non-inflammatory lesions displayed a more prompt response, ascertaining the supremacy of adapalene in the treatment and maintenance of lessening comedonal acne. The efficacy of adapalene 0.1% and 4% benzoyl peroxide combined have shown similar trends in a multi-central study done on a higher Fitzpatrick scale skin population by Dréno B et al. [14].

Interestingly, a very limited number of international studies have compared adapalene 0.1% and 4% benzoyl peroxide as monotherapies in the SOC population. More importantly, when seen from a national perspective, our study strengthens the current knowledge by adding to the limited amount of research expeditions in Pakistan regarding the comparative analysis of these two important therapeutic modalities in the skin SOC population. This could help shape a final consensus regarding the efficacy of these two agents. As per our findings, Benzoyl peroxide was efficacious in 75% of the subjects, comparable to the local and international investigations conducted on the SOC population [15,16]. However, in contrast to a study by Dubey and Amane [16], our study found no difference in the efficacy of 0.1% adapalene and 4% benzoyl peroxide [15,16]. In the follow-up term, benzoyl peroxide demonstrated a prompt deduction of inflammatory lesions identical to other investigations. In the comparison of the efficacy of adapalene and benzoyl peroxide in the SOC population, both were efficacious in the deduction of lesions at week 12 with no substantial difference (p>0.05). This observation is similar to findings of other global research conducted on fair-skinned populations, where a comparison of the two agents independently or in combination demonstrated no substantial dissimilarity in their effectiveness [17].

As far as the side effects of 0.1% adapalene and 4% benzoyl peroxide are concerned, the safety profile of adapalene demonstrated that dryness was the main side effect (in 68% of patients). Other adverse effects included burning (53%) and erythema (22%), similar to previous investigations [17]. With repeated application, the dryness and erythema thanks to adapalene were subsequently abated. This “period of sensitization” has also been noted in the research performed on lower Fitzpatrick skin types and the adverse effects largely subsided after 12 weeks of application [16]. The adverse events reported with benzoyl peroxide were burning and dryness which can be attributed to the keratolytic qualities of this medicine but the main unfavourable effect with this agent was reported to be erythema. Identical adverse effects have been reported by different earlier studies as well, although the proportion in our study was lower compared to previous studies [16,17]. However, none of the side effects led to discontinuation of either drug in our study population and both drugs were largely well tolerated in our SOC cohort, with one medication failing to demonstrate supremacy over the other in terms of side effects and tolerability, in contrast to a recent Indian study [16]. 

BMI was calculated in both groups to ensure there was minimal bias and also to limit BMI's ability as a factor that could alter the effects of both drugs in each group. since the mean BMI was comparable in both groups, we argue that this factor was neutralized. We excluded high BMI patients from our study as high BMI may affect acne treatment outcomes.

Limitations

The limitations of our study include a smaller sample size and our inability to perform a direct comparison between different skin types (Fitzpatrick type I & II vs type III and higher) due to a lower turnout of fair-skinned individuals to our dermatology clinic. We used previous studies done on fair skin populations as references to determine the comparison between 0.1% adapalene and 4% benzoyl peroxide. Since our study caters to the SOC population, using a study conducted on fair-skinned individuals for our sample calculation may have affected our results. A family history of acne among the participants could not be recorded during the research. Although we did compare the two drugs for their efficacy and recorded their salient side effects in our patients, we didn't do a proper statistical comparison of the side effects of adapalene and benzoyl peroxide. Further large-scale multi-center studies may be needed to determine any difference in the safety and efficacy of these drugs in different skin types simultaneously.

## Conclusions

Our study concludes that 0.1% adapalene is as effective and safe as 4% benzoyl peroxide in the skin of color (SOC) population. Both of these options are very effective in treating mild to moderate acne vulgaris by curbing bacterial colonization and the inflammatory process in the SOC population. Benzoyl peroxide is, nonetheless, efficacious in the earlier deduction of inflammatory lesions (papules and pustules) while adapalene is more effective in the reduction of non-inflammatory (comedonal) lesions and the maintenance therapy in such patients.

## Figures and Tables

**Figure 1:**
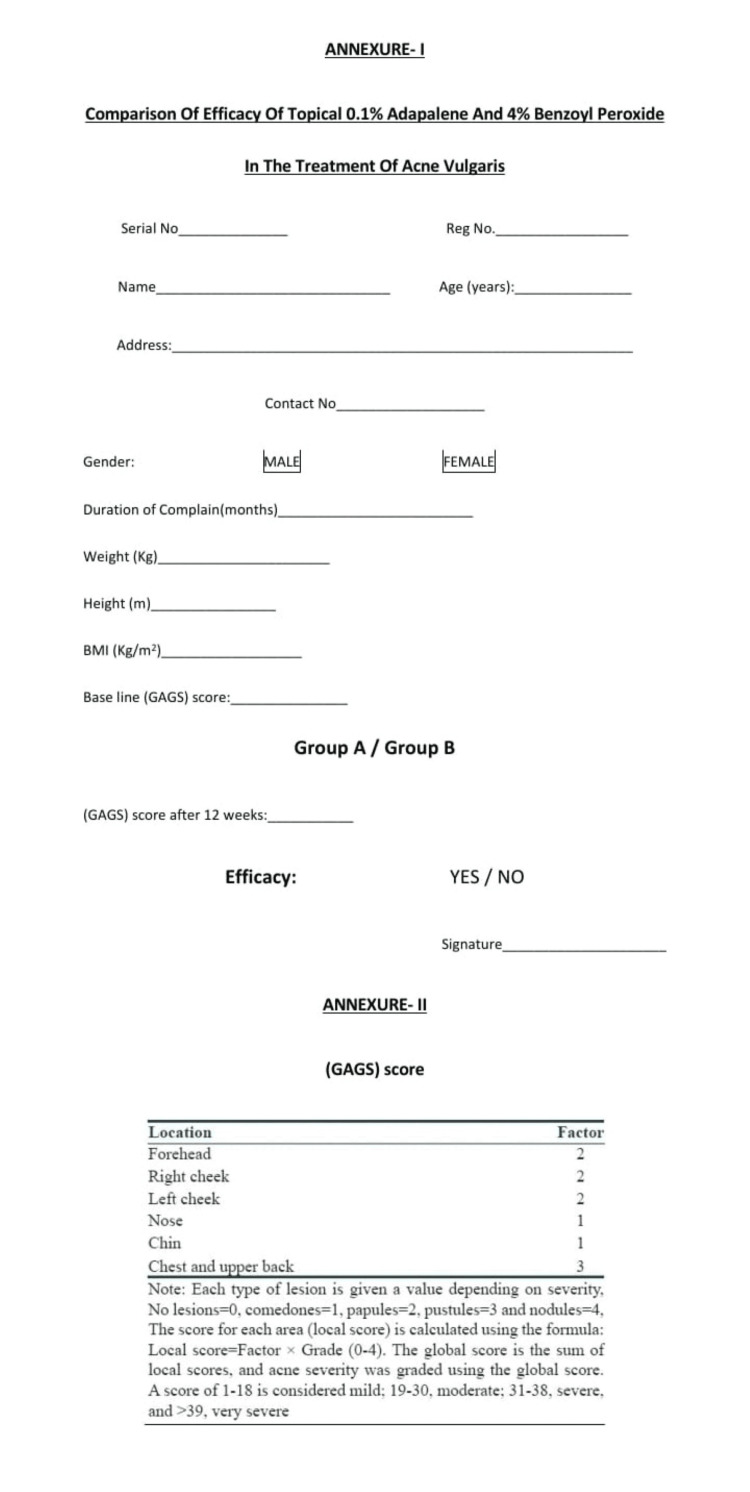
Specially designed proforma for data collection
